# On the potential of *in vitro* organ-chip models to define temporal pharmacokinetic-pharmacodynamic relationships

**DOI:** 10.1038/s41598-019-45656-4

**Published:** 2019-07-03

**Authors:** Christopher W. McAleer, Amy Pointon, Christopher J. Long, Rocky L. Brighton, Benjamin D. Wilkin, L. Richard Bridges, Narasimham Narasimhan Sriram, Kristin Fabre, Robin McDougall, Victorine P. Muse, Jerome T. Mettetal, Abhishek Srivastava, Dominic Williams, Mark T. Schnepper, Jeff L. Roles, Michael L. Shuler, James J. Hickman, Lorna Ewart

**Affiliations:** 1 Hesperos, Inc., 3259 Progress Dr., Room 158, Orlando, FL 32826-3230 USA; 2 Drug Safety and Metabolism, IMED Biotech Unit, AstraZeneca, Cambridge, UK; 3 Drug Safety and Metabolism, IMED Biotech Unit, AstraZeneca, Waltham, USA; 4 NanoScience Technology Center, 12424 Research Parkway, Suite 400, Orlando, FL 32826 USA

**Keywords:** Toxicology, Pharmacokinetics

## Abstract

Functional human-on-a-chip systems hold great promise to enable quantitative translation to *in vivo* outcomes. Here, we explored this concept using a pumpless heart only and heart:liver system to evaluate the temporal pharmacokinetic/pharmacodynamic (PKPD) relationship for terfenadine. There was a time dependent drug-induced increase in field potential duration in the cardiac compartment in response to terfenadine and that response was modulated using a metabolically competent liver module that converted terfenadine to fexofenadine. Using this data, a mathematical model was developed to predict the effect of terfenadine in preclinical species. Developing confidence that microphysiological models could have a transformative effect on drug discovery, we also tested a previously discovered proprietary AstraZeneca small molecule and correctly determined the cardiotoxic response to its metabolite in the heart:liver system. Overall our findings serve as a guiding principle to future investigations of temporal concentration response relationships in these innovative *in vitro* models, especially, if validated across multiple time frames, with additional pharmacological mechanisms and molecules representing a broad chemical diversity.

## Introduction

Integrative pharmacology is a discipline that builds an understanding of the inter-relationship between pharmacokinetics (PK), the drug’s time course for absorption, distribution, metabolism and excretion and pharmacodynamics (PD), the biological effect of a drug. In drug discovery, this multi-variate approach guides medicinal chemists to modify structural properties of a drug molecule to improve its chance of becoming a medicine in a process known as “lead optimization”. Pharmacological effect is driven by drug concentration however the seminal work of Segre^1^ and Sheiner *et al*.^2^, identified that the maximum drug effect is not always driven by the peak drug concentration but that in some cases time is a critical factor influencing drug effect, especially in *in vivo* studies. This is known as hysteresis and is driven by limited access to the site of drug action or slow receptor kinetics. In the main, current drug discovery programs are only able to truly investigate concentration-effect-time relationships during the advanced stages of the preclinical program. At this stage there are typically between 1 and 3 potential drug candidates progressed to animal studies to confirm efficacy and safety. It is therefore costly and time consuming to discover that such potential candidates may have poor therapeutic qualities preventing their onward progression, not to mention the significant limitations of extrapolating animal data to humans because drug behavior in animals can be dramatically different to that in humans.

Over the last two decades there has been an explosion in the development of microphysiological systems (MPS) or “body-on-a-chip” models^3,4^. These microengineered *in vitro* models aim to recreate the *in vivo* tissue microenvironment enabling cells, typically human in origin, to maintain viability and function. The hype that these systems could recapitulate organ function^5^ is gradually being replaced by hope that such systems might enable more eloquent models for drug discovery reducing the reliance on animals which has ethical, monetary, time and translational advantages.

Using a ‘heart-on-a-chip’ that was fluidically connected to a ‘liver-on-a-chip’ we set out to discover whether it was possible to derive *in vitro* temporal PKPD relationships with the well characterized small molecule drug terfenadine. This multi-organ platform utilized a pumpless system and serum-free medium with interconnected compartments^6,7^. The absence of a pump and the accompanying tubing enabled a bubble free, low volume system providing the ability to detect metabolite formation that might otherwise be diluted in higher-volume systems^8^. The sinusoidal oscillatory rocking profile for driving fluid flow was designed via a transient flow model driven by gravitational acceleration to produce shear stresses on the cellular layers of no more than 0.05 dynes/cm^2^ (within acceptable physiological ranges)^8,9^ throughout the rocking profile, as described previously^6^. This PK model additionally includes parameters for convective transport of compounds throughout the system (mixing) and compound-specific adsorption to the housing materials and has been used for both hydrophobic and hydrophilic compounds^7^. Currently, the adsorption is determined experimentally for each compound, though this framework is expected to be expanded to create a prediction of the adsorption based on the physical characteristics of the compounds. This PK model is used to inform our predictive PKPD model and the effect of metabolites on toxicity in multi-organ systems has also been explored at single timepoints in this model^10,11^. The functional cardiac system utilized patterned human cardiomyocytes that had previously been shown to allow non-invasive measurements of field potential duration (FPD), conduction velocity and beat rate^12^.

We reproduced the terfenadine-induced increase in QT-interval in the heart only model and demonstrated that in the presence of a metabolically competent liver compartment, this effect was diminished. We also discovered that this pharmacodynamic effect was driven by the intracellular drug concentration in cardiomyocytes, consistent with the intracellular binding site of terfenadine to the hERG channel^13,14^. Furthermore, we have been able to build a mathematical model that predicts drug response in this MPS model and additionally that these approaches can also be used to qualitatively predict the effect of terfenadine in preclinical species. Finally, we demonstrated the value of MPS models in drug discovery by testing an AstraZeneca proprietary small molecule which was identified as having a hERG liability, due to the formation of a hERG active metabolite, during the final stages of lead optimization. Taken together, these results provide the first description of integrative pharmacology in MPS models and builds confidence towards the transformative effect these models can have on drug discovery.

## Results

### Characteristics of heart:liver and heart only MPS models

**Figure 1:**
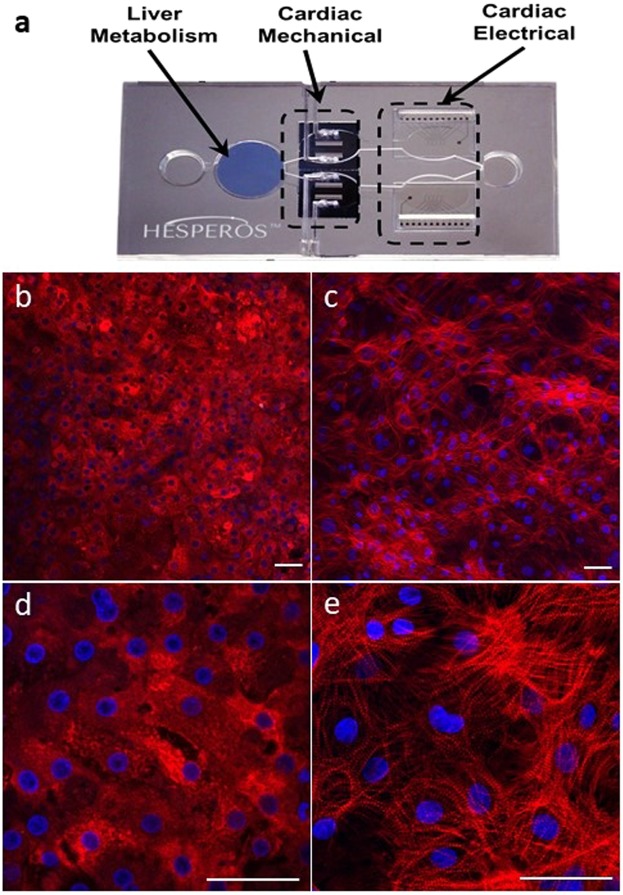
Overview of the heart only and heart:liver MPS models; (**a**) Photograph of the MPS platform showing the positioning of the 3 cellular chambers (1 containing hepatocytes and 2 containing hiPS-CMs) and integrated technology for assessment of cardiac mechanical and electrical activity interconnected via a closed loop recirculating flow system. (**b**,**d**) Confocal immunofluorescent images showing primary hepatocytes stained for albumin (red) and nuclei (blue), clearly indicating the cytoplasmic localization. (**c**,**e**) Confocal immunofluorescent images showing hiPS-CMs stained for myosin heavy chain (red) and nuclei (blue), indicating the presence of aligned sarcomeres. Scale bars in white are 50 µm. All images are representative of at least 6 biological and at least 40 technical replicates.

Figure 1 shows the composition of the heart:liver and heart only models. Each model encompassed a microfluidic system (total volume 2 mL), 2 media chambers and 2 (heart model) or 3 (heart:liver model) cellular chambers (1 liver and 2 cardiac). These cellular chambers contain electrodes (multi electrode array; MEA) and flexible cantilevers to assess cardiac electrical and mechanical function, respectively (Fig. 1a). Only the MEA cardiac chamber was utilized in this study. Following cell attachment with human primary hepatocytes in the liver chamber and patterned human induced pluripotent stem cell derived cardiomyocytes (hiPS-CMs) in the heart chamber, each model was placed on an interval rocker that induced flow by reciprocal levelling between reservoirs resulting in a closed loop recirculating flow^12^. This recirculating serum-free system supports the maintenance of liver (cytochrome (CYP) P450 activity and albumin production) and cardiac function for up to 28-days^15^. The presence and cellular structure of key hepatocyte (albumin) and cardiomyocyte (myosin heavy chain) markers were confirmed following 14 days by immunofluorescence (Fig. 1b–e). Cardiomyocyte resting membrane potential was shown to be physiologically relevant at approximately −85 to −90 mV, representative of the adult human heart^16–18^. Taken together this provides evidence that the heart:liver model is a suitable system to investigate both parent compound and metabolite driven cardiovascular changes. Importantly the integrated technology also allows *in vitro* temporal PKPD relationships to be derived.

### Value of temporal PK and FPD measurements of parent compound driven changes

To build insight into whether an MPS model could be used to derive temporal PKPD relationships, the well-characterized small molecule, terfenadine was utilized. Terfenadine is a hERG inhibitor resulting in QT prolongation in both preclinical species and in humans, but it is rapidly metabolized, mainly by CYP3A4, to fexofenadine which does not cause QT prolongation^19,20^. The integrated MEA electrodes in both the heart only and heart:liver models were utilized to measure the external field potential. This allows the quantification of FPD, a surrogate of the action potential duration at 90% repolarization (APD_90_), hence enabling an assessment of the impact of terfenadine on the QT interval^21^.

**Figure 2:**
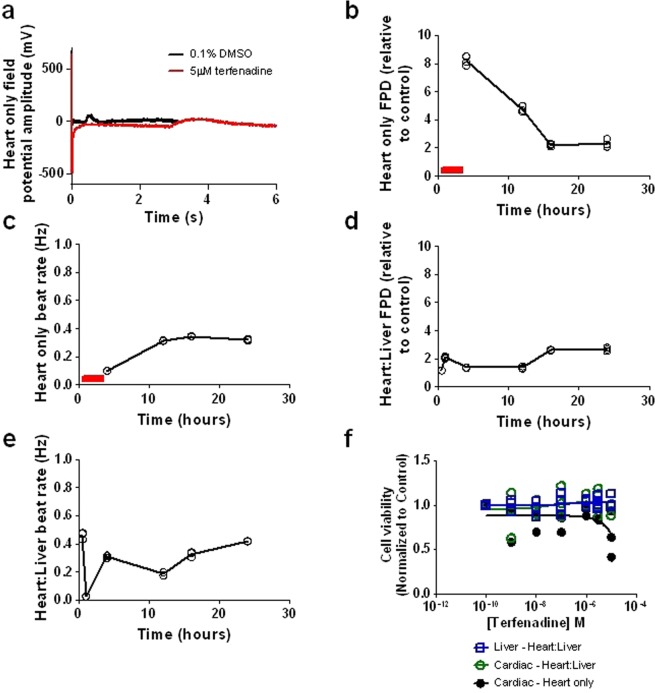
Terfenadine induced PD response in the heart only and heart:liver models; (**a**) representative field potential traces following 5 µM terfenadine (red) or vehicle control (0.1% DMSO) (black) at 4 h in the heart only system. (**b**) Quantification of FPD and (**c**) beat rate in the heart only model, 30 second measurements were taken at 0, 0.5, 1, 4, 12, 16 and 24 hours, the solid line is the mean of 3 replicates with individual data points plotted. The red bar represents timepoints where no FPD could be quantified due to the arrhythmogenic effects of terfenadine. (**d**,**e**) Quantification of FPD and beat rate in the heart:liver model, 30 second measurements were taken at 0, 0.5, 1, 4, 12, 16 and 24 hours, the solid line is the mean of 3 replicates with individual data points plotted. (**f**) Cell viability in both the cardiomyocyte and hepatocyte cellular chambers in both the heart only (black) and heart:liver models (heart: green; liver: blue) following 24 hours of terfenadine, individual data points plotted. All data n ≥ 3.

Heart:liver and heart only models were exposed to 5 µM terfenadine or vehicle (0.1% DMSO). Simultaneous heart FPD, beat rate, terfenadine and fexofenadine concentrations in the media and cellular lysates were measured at 0, 0.5, 1, 4, 12, 16 and 24 hours. Figure 2a depicts typical field potential traces obtained from the heart only model. Using these traces FPD and beat rate were quantified, as represented in Fig. 2b–e in the heart only (Fig. 2b,c) and heart:liver model (Fig. 2d,e). In the heart only model, time dependent increases in FPD were noted, with peak measurable changes (8-fold increase) observed at 4 hours. A 5 µM nominal concentration of terfenadine in heart only systems had a profound effect on cardiac repolarization at 0.5 to 1 hour that rhythmic cardiac beating ceased and therefore, FPD (Fig. 2b) and beat rate (Fig. 2c) were unquantifiable. These changes in FPD were reduced in the heart:liver model, over the time course of the experiment (Fig. 2d). This data provides evidence that the FPD responses observed were driven by terfenadine (the parent drug). No changes in cell viability in either the heart or liver chambers at 24 hours were present in either model (Fig. 2f), confirming these changes in FPD were not the result of gross cell death.

**Figure 3:**
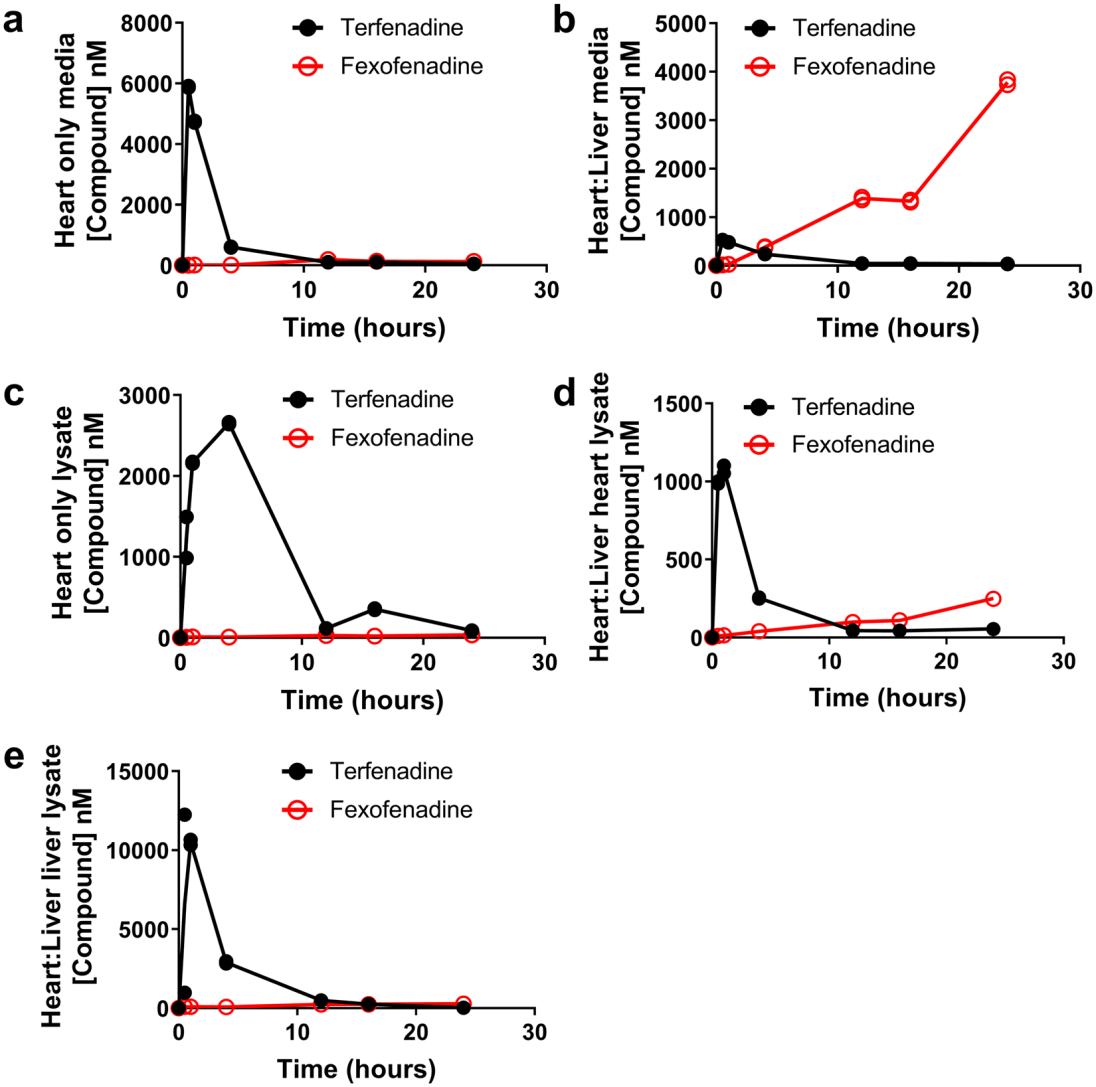
Terfenadine induced PK response in the heart only and heart:liver models. Both terfenadine (black) and fexofenadine (red) concentrations where quantified in the media and cellular lysates following 5 µM terfenadine; measurements were taken at 0, 0.5, 1, 4, 12, 16 and 24 hours, the solid line is the mean of 2 replicates with individual data points plotted. (**a**,**b**) PK profiles in media from the heart only (**a**) and heart:liver (**b**) models. (**c**–**e)** PK profiles in cellular components from the heart only (**c**) and heart:liver heart lysates (**d**); (**e**) liver lysates MPS models.

Further evidence that the differences in FPD observed between the heart only and heart:liver models following terfenadine were due to a linked metabolically competent liver, a temporal PKPD relationship was derived by measuring both terfenadine and fexofenadine concentrations in the recirculating media and in cellular lysates of the cardiomyocytes and hepatocytes (Fig. 3). In media from the heart only model, terfenadine concentration also peaked at 0.5 hours, but higher concentrations were measured, 5.88 µM ± 0.032 µM (mean ± SD) and there was limited metabolism to fexofenadine (0.124 µM ± 0.003 µM; mean ± SD) (Fig. 3a). In the heart:liver model, the terfenadine concentration peaked at 0.5 hours at 0.53 µM ± 0.002 µM (mean ± SD) while the fexofenadine concentration increased over the 24 hour measurement period in a time-dependent manner, achieving 3.8 µM ± 0.075 µM (mean ± SD) at 24 hours (Fig. 3b). This confirms that metabolism of terfenadine occurred when hepatocytes are included. However, loss of terfenadine from media samples was observed by 4 hours in the heart:liver model. This highlights a disconnect between the terfenadine media concentration and FPD response in the heart only model. For example, at the 12-hour timepoint, FPD was increased by 4.8-fold, but the media terfenadine concentration was only 0.099 µM. At similar concentrations, but later timepoints in the heart:liver model, no notable increase in FPD was observed. To understand this apparent disconnect between the media terfenadine concentration and cellular pharmacodynamic response, cellular lysate concentrations of terfenadine and fexofenadine in each individual cell chamber were assessed (Fig. 3c–e). Within cardiomyocyte lysates from the heart only model, the terfenadine concentration peaked at 4 hours (2.65 µM ± 0.016 µM; mean ± SD) and decreased over the measurement period which corresponded to the decrease in FPD measured (Fig. 3c). In the cellular chambers of the heart:liver model, terfenadine concentrations peaked between 0.5 and 1 hours, with the accumulation in the liver chamber, 12.04 µM ± 0.28 µM (mean ± SD), being 10-fold higher to that in the heart chamber, 1.08 µM ± 0.04 µM (mean ± SD) (Fig. 3d–e). Taken together this indicates that the terfenadine-induced change in FPD was driven by the cellular cardiomyocyte concentration (Supplementary Fig. 1). In addition, to the best of our knowledge, this is the first time that an MPS model has been used to derive a quantitative temporal PKPD relationship.

### Qualitative and quantitative translational modelling

Qualitatively the terfenadine response was comparable across standard preclinical and clinical cardiovascular safety studies (Table 1). It is important to note that quantitative differences in the magnitude of QT prolongation across pre-clinical species and human is known, the occurrence of QT prolongation can be qualitatively compared, but predicting the magnitude of change from either Guinea pig or dog pre-clinical studies is variable. For example, dogs have been found to be approximately 2.5 times less sensitive to QT prolongation in terms of the magnitude of response^22^.

**Table 1:** Qualitative comparison of the effects of terfenadine in the MPS systems compared to conventional approaches applied during early drug discovery to detect changes in QT prolongation^23,24,55–57^.

Model	Assay/endpoints	Result	Dosing Scheme	Time Frame	Reference
Heart only MPS	FPD	+4.4% at 1.912 μM	Nominal dose at time 0	Average observed data over 24 hours	—
Heart:Liver MPS	FPD	+1.9% at 0.228 μM	Nominal dose at time 0	Average observed data over 24 hours	—
CHO cells overexpressing hERG/Kv11.1	hERG inhibition (electrophysiology)	IC_50_ 0.02–0.2 μM	Non-cumulative concentration response curve	3–5 minutes	Redfern *et al*.^57^
Anaesthetised Guinea pig	QTc	+8% at 0.0384 μM	IV infusion for 10 minutes	Peak measurements post infusion (10–40 minutes)	Yao *et al*.^23^
Anaesthetised Guinea pig	QTc	+2.8% at 0.0069 μM	IV infusion for 10 minutes	Peak measurements post infusion (10–40 minutes)	Yao *et al*.^23^
Dog Telemetry	QTc	+2.34% at 0.026 μM	IV infusion over 180 minutes	Average measurements from 150–180 minutes	Ollerstam *et al*.^24^
Dog Telemetry	QTc	+1.72% at 0.0078 μM	IV infusion over 180 minutes	Average measurements from 150–180 minutes	Ollerstam *et al*.^24^
Human	QTc	+1.5% at approx 0.004 μM	2X daily 60 mg dose orally	QTc measured day 1–5	Chen^56^; Pratt *et al*.^55^

**Figure 4:**
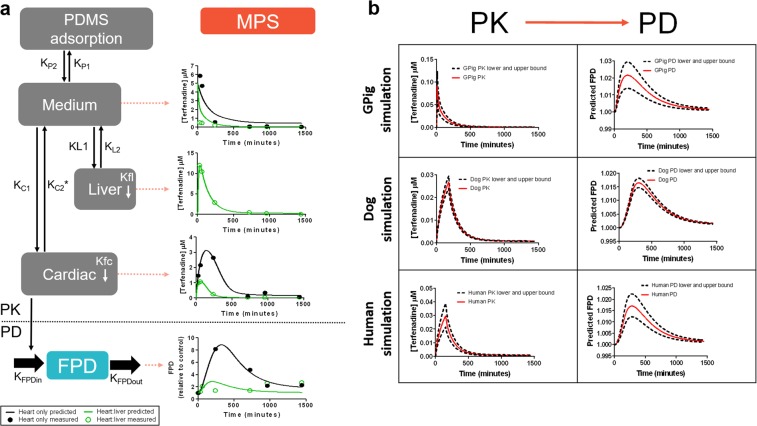
PKPD modelling for the heart only and heart:liver models. (**a**) Schematic of the modelled system is shown where PDMS, cardiac, and liver compartments allow flow of terfenadine to and from the media compartment. Cardiac and liver compartments are capable of metabolizing terfenadine into fexofenadine and change in FPD is solely dependent on cardiac terfenadine concentration. Terfenadine PK and FPD profiles are plotted for predicted and observed data over 24 hours and are modelled in both heart only and heart:liver systems. Corresponding fexofenadine PK profiles can be found in Supplementary Fig. 2. *Parameter is defined by Eq. 2. (**b**) Published^21,22^, mean Terfenadine PK profiles ± SD for Guinea pig, dog and humans were inputted into the model and corresponding mean FPD predicted values ± SD were simulated.

Due to the measurement of both the PK and PD response simultaneously, a PKPD relationship was developed to predict the FPD changes obtained in these models. To develop a quantitative translational model, temporal data across the MPS platform for terfenadine, fexofenadine, and FPD measurements were used to develop a mathematical PKPD model using Phoenix NLME 7.0 software. The mathematical model could accurately capture terfenadine and fexofenadine exposure levels in the MPS model over time (Fig. 4a and Supplementary Fig. 2). It was further able to capture changes in FPD signals produced by the heart chamber (Fig. 4a). Corresponding peak shifts in cardiac terfenadine PK profiles and FPD profiles verify the assumption that changes in FPD are associated with cardiac bioaccumulation of terfenadine. The goodness of fit captured by the model parameter estimates was further reinforced through percent coefficient of variation (% CV) rates below 26% (Supplement Table 1).

FPD simulations were performed based on published *in vivo* PK profiles to demonstrate the potential of the MPS model to predict cardiotoxicity. Representative PK profiles for Guinea pig^23^, dogs^24^ and human^25^ were used as an input for the simulation to predict corresponding cardiac terfenadine bioaccumulation and therefore predicted FPD profiles (Fig. 4b). The predicted FPD values were qualitatively comparable to the reported dog and human QTc prolongation seen in Table 1; however, the FPD predictions failed to reach published Guinea pig QTc prolongation levels (Table 1). The quantitative translation is likely to have been limited by several potential sources of variability; (1) differences between *in vitro* and *in vivo*: the chip is made from human cells, and although stem-cell models are commonly used to explore FPD, there remains a possibility that the exact response magnitude differs between the chip and *in vivo*; (2) donor to donor variability has not been accounted for and could potentially be a factor affecting the quantitative responses measured; (3) variability between species: there are clearly different responses observed *in vivo* between different species and to address the impact of this would require development and validation of species-relevant chip models which is beyond the scope of this manuscript and (4) variability between *in vivo* responses: the *in vivo* models do not all agree from study to study, and different observed exposure profiles and FPD could be another confounding layer in the interpretation of the results. Nonetheless, these *in vivo* simulations show the potential for the MPS model to qualitatively predict cardiotoxicity in humans and in animal models, especially the dog and could be improved to reach quantitative predictions by reducing sources of variability.

### Application in drug discovery

**Figure 5:**
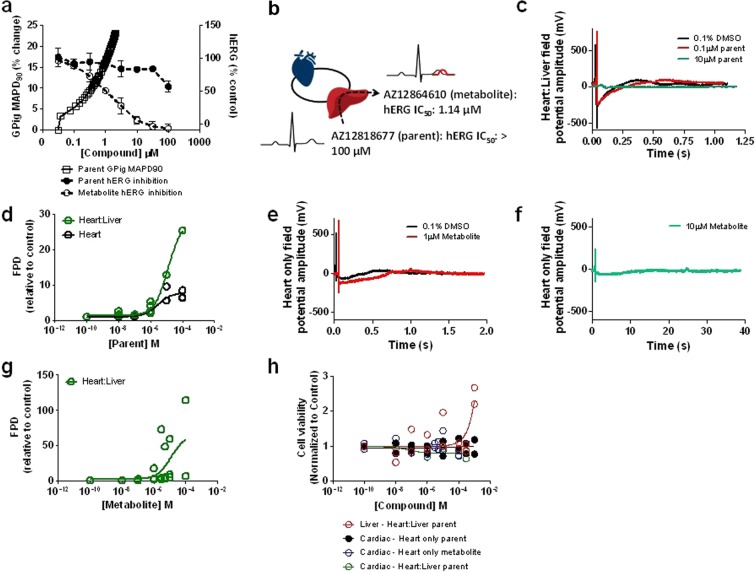
‘Real-world’ application of heart:liver models to detect metabolite driven PD effects. (**a)** Overview of the preclinical *in vitro* and *in vivo* QT data for both the parent compound (AZ12818677) and its metabolite (AZ12864610), concentration-effect curves for hERG activity and *in vivo* QT prolongation in the Guinea pig, n ≥ 3. (**b**) Schematic of AZ12818677 (parent) and AZ12864610 (metabolite) effects on QT prolongation. (**c)** Representative field potential traces following 0.1% DMSO (black), 10 µM (green) and 0.1 µM (red) of the parent compound in the heart:liver model. (**d**) Concentration-effect curves of the parent compound FPD response in both the heart only (black) and heart:liver (green) models following 24 hours treatment, n ≥ 3 (in the heart:liver model at concentrations ≥30 µM, arrhythmic events were observed in two out of three replicates). (**e**) Representative field potential traces following 0.1% DMSO (black) and 1 µM (red) of the metabolite in the heart only model. (**f**) Representative field potential traces following 10 µM (green) of the metabolite in the heart only model. (**g**) Concentration-effect curve of the metabolite FPD response in the heart only model, arrhythmias were observed at concentrations ≥ 3 µM, n ≥ 3. (**h**) Cell viability in both the heart and liver cellular chambers in both the heart only (parent: black; metabolite: blue) and heart:liver models (heart: green; liver: red) following 24 hours, n = 2. Individual data points plotted.

Application of MPS model data to build further confidence in the human risk of industrial scientists involved in drug safety disciplines of pharmaceutical companies. Following the characterization of the MPS model with terfenadine and a proprietary AstraZeneca compound (AZ12818677) was evaluated in both the heart only and heart:liver model. Prolongation of the QT interval on the electrocardiogram can lead to a fatal arrhythmia known as Torsade de Pointes^26^. Consequently, it is a mandatory requirement prior to first in human administration of potential new drug candidates that their ability to inhibit the hERG channel, a biomarker for QT prolongation, is investigated. This is typically conducted *in vitro* at a very early stage in drug discovery because of high-throughput cellular technologies^27^. During the lead optimization phase of drug discovery this *in vitro* data is supplemented by an *in vivo* assessment in small animals, typically either the Guinea pig or rat, providing an integrated assessment of cardiovascular safety. These studies allow compounds to be profiled while chemical diversity is available, facilitating the selection of optimal molecules prior to mandated regulatory evaluation of cardiovascular function in non-rodents. AZ12818677 (parent compound) was assessed for hERG inhibition as is standard practice and no quantifiable IC_50_ value could be derived at concentrations up to 100 µM. It was concluded that this compound had a low potential to cause *in vivo* QT prolongation. AZ12818677 was therefore progressed to a small animal cardiovascular *in vivo* study to assess QT prolongation, in this case the anaesthetized Guinea pig. However, unexpectedly the corrected QT interval increased by 22% (Fig. 5a) at a concentration of 3 µM. Due to the lack of activity of AZ12818677 at the hERG channel, it was concluded that the QT prolongation in the Guinea pig was not being driven by AZ12818677-induced hERG inhibition. Subsequent investigations identified a major metabolite, AZ12864610, which was produced in humans and Guinea pig (among other preclinical species). Retrospective analysis of AZ12864610 on the *in vitro* hERG assay, revealed it was a potent inhibitor with an IC_50_ of 1.14 µM (95% confidence interval (CI) 0.86 µM – 2.2 µM). Due to this metabolite driven effect on hERG activity and the *in vivo* QT prolongation we investigated whether the heart:liver model could prospectively detect this metabolite driven effect (Fig. 5b).

Figure 5c highlights representative FP traces following the parent (AZ12818677) compound in the heart:liver model at 24 hours. These traces were quantified, resulting in an EC_50_ of 2.7 µM (95% CI 0.9–7.6 µM) and 13.2 µM (95% CI 9.8–17.9 µM) on FPD in the heart:liver and heart only models, respectively (Fig. 5d). To confirm the changes in the FPD in the heart:liver model were a result of the formation of the metabolite AZ12864610, it was chemically synthesized and evaluated in the heart only model. As expected, the FPD was prolonged resulting in an EC_50_ of 1.2 µM (95% CI 0.7–1.9 µM) (Fig. 5g). At concentrations above 3 µM, there were incidents of extreme FPD prolongation (Fig. 5e–f) to the point of arrhythmogenesis suggesting a profound effect on the electrical activity of the cells. This arrhythmogenic activity resulted in large error associated with FPD measurements which were removed from the graphical representation for the sake of clarity (Fig. 5f), but clearly a pharmacodynamic effect was detectable. In addition, cell viability in either the heart or liver chambers at 24 hours was not reduced (Fig. 5h), confirming these changes are not the result of gross cell death. The FPD data obtained for these AstraZeneca compounds and terfenadine demonstrate that these MPS model systems can detect cardiovascular changes driven by either the parent molecule or the metabolite.

**Figure 6:**
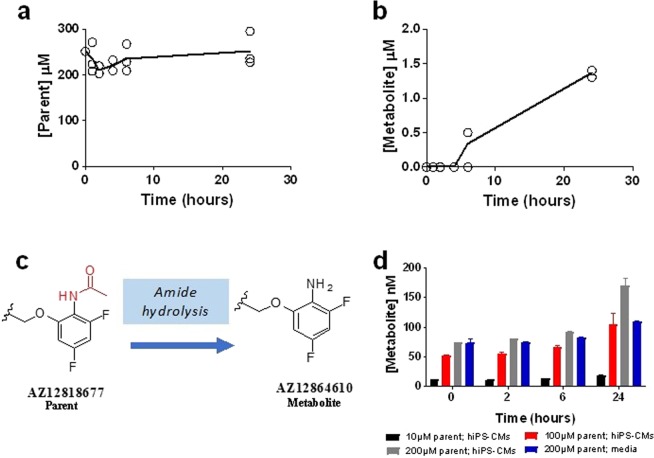
Metabolism of AZ12818677 to AZ12864610. (**a**,**b**) Both parent (AZ12818677) (**a**) and metabolite (AZ12864610) (**b**) concentrations where quantified in media from the liver compartment following 300 µM parent; measurements were taken at 0, 1, 2, 4, 6 and 24 hours, the solid line is the mean of 3 replicates, with individual data points plotted. (**c)** Metabolic reaction representing de-acetylation/amide hydrolysis of AZ12818677 (parent) to form AZ12864610 (metabolite). (**d**) Determination of AZ12864610 (metabolite) concentrations at different time points in cardiomyocytes/media following incubations with AZ12818677 (parent).

The ability of the liver cellular component to metabolize AZ12818677 to AZ12864610 was assessed. The measured concentration of AZ12818677 remained relatively stable over 24 hours. For example, following a nominal concentration of 300 µM, 251 µM (SD 0 µM) was measured in the media of the liver component at 0 hours, and 251 µM (SD 5.7 µM) was measured at 24 hours (Fig. 6a). Associated with this, only 0.5 µM and 1.4 µM of AZ12864610 (the metabolite) was detected at 6 and 24 hours respectively (Fig. 6b). Taken together this suggests a complex PKPD relationship, with additional factors influencing the PK and metabolism of the parent compound. Due to the presence of a PD effect in the heart:liver and heart only model despite minimal concentrations of the metabolite, we investigated the potential of cardiomyocytes to produce the metabolite. Since this metabolite is a product of amide hydrolysis (Fig. 6c) it is possible that cardiomyocytes can produce it through cholinesterases^28^. Formation of this metabolite in cardiomyocytes (Fig. 6d) is a significant observation which confirms the PD response in the cardiac only system is derived from a metabolite formed by cardiomyocytes and the response observed in heart:liver model could be a cumulative effect. Although we did not investigate if AZ12864610 undergoes an N-acetylation reaction to form AZ12818677, it could be a possible explanation for the limited detection of the metabolite in incubations with hepatocytes (Fig. 6b), which can potentially convert the metabolite back to parent via N-acetyl transferase enzymes.

## Discussion

Human-on-a-chip models are gaining increasing popularity as tools that will improve preclinical translation thus ensuring potential new drug candidates have a higher probability of success during the clinical trial process. Indeed, the pharmaceutical industry have pointed to defining their context of use to realize their full impact^29^. But to date, these models have been largely characterized only with well-studied reference compounds and so the performance of these models in a ‘real-world’ setting remains to be determined^30,31^. Moreover, little attention has been paid to exploiting the dynamic nature of these models to derive an understanding of the temporal relationship between measured pharmacodynamic response and pharmacokinetics^30^. The ability to build PKPD relationships *in vitro* and associated quantitative translational understanding would enable scientists to understand compound behavior prior to *in vivo* testing, offering a cost and time saving as well as a credible option to reduce and eventually replace animal models. Furthermore, although several groups have developed computational models to analyze data from MPS models^7,8,32,33^, no-one has yet published data on quantitative translation of MPS data to *in vivo* outcome whether that be in animals or humans^34^. Here we report first on the use of a heart-liver MPS model to develop a temporal PKPD relationship for the anti-histamine, terfenadine. Using the quantitative data, we then show that we can build a mathematical model that correctly predicts the PK and PD in the MPS, accounting for parent and metabolite concentration across compartments of the device (e.g. conservation of mass) as well as the relationship between target tissue exposure and pharmacodynamic outcomes. Because the model allows the PK and PD responses to be evaluated separately, we were then able to compare the *in vitro* PD response with *in vivo* outcome in commonly used preclinical species. Finally, we demonstrated the application of this MPS model to drug discovery by recreating the parent and metabolite effects of a proprietary AstraZeneca small molecule compound, which in contrast to terfenadine the cardiovascular effects were driven by the metabolite.

Our simultaneous measurement of cardiomyocyte response to the cardiotoxic drug terfenadine, together with matched quantification of drug concentration in the circulating media and in the lysates of both cardiomyocytes and hepatocytes, show that the pharmacodynamic response was not driven by the concentration of drug in the media but rather by the concentration of drug within the cardiomyocytes. This was also supported by the lack of response of cardiomyocytes to terfenadine in the presence of metabolically competent hepatocytes and the time-dependent increase in the concentration of the hERG-inactive metabolite fexofenadine in the circulating media. Although it is known that terfenadine binds to an intracellular site of hERG^13,14^, we have for the first time in a dynamic *in vitro* system convincingly proven this^35^. With this knowledge, we can determine the peak effect of terfenadine in the model, rather than at a fixed time point^36^. This principle is fundamental to developing an understanding of drug behavior and being able to define this during early drug discovery will be a valuable contribution to the optimization of potential new drug candidates.

The commitment to measure concentration and effect over time enables an accurate description of drug behavior^37^ and is a critical component of quantitative translation. Most of the current *in vitro* literature assumes that the concentration of drug added to the media drives the effects measured^38^ and that the administered concentration is equivalent to the effective concentration. It has been established that polydimethylsiloxane (PDMS), commonly used in MPS models, absorbs drug compounds, especially those that are highly lipophilic^39^ and measuring actual drug concentration also addresses these limitations. As demonstrated with terfenadine, the PKPD modelling approach was critical for understanding both the flux of compound between compartments as well as the resulting PD response in the context of dynamic exposure profiles of parent and metabolite. Further evaluation of a proprietary AstraZeneca small molecule compound highlights the complexity of changes observed with novel molecules during drug discovery, providing an example of a ‘real-world’ application. This serves as an illustration of the complexity and diversity of PKPD relationships different compounds can drive. This diversity and ability of both PK and PD effects to occur through both direct and indirect interactions, either instantaneously or delayed further highlights the importance of assessing both the PD and PK effects in conjunction with each other to allow multiple influences on hysteresis to be evaluated^40^.

The application of mathematical modelling of cardiovascular safety data is well established in terms of PKPD modelling of *in vivo* data to predict and translate human effects at expected therapeutic doses^41^. However, the use of these modelling approaches to MPS data is still in its infancy but has been shown to enhance the value of the MPS models^7,42–44^. Here we demonstrate the potential to use mathematical modelling for translational assessment in the same well-established manner as is commonly applied to *in vivo* study data. Just as with translating *in vivo* data across species, the model-derived PK profile and PKPD relationships are built and extrapolated to *in vivo* scenarios for a single compound at a time by substituting exposure profiles. Further, as with translational validation of *in vivo* models, proof of concept is initially established with a small number of compounds and confidence in the translational utility of the experimental platform will grow as additional data is generated across differential chemistry, pharmacology and mechanisms. This joint experimental-computation approach provides the prospect of creating quantitative translational understanding for cardiovascular changes based on a human model that can be applied within drug discovery, predicting the preclinical and clinical changes.

Within this study we have assessed one element of cardiovascular safety: changes in ECG waveforms and intervals. However, drug-induced cardiovascular toxicity can also result in modulation of hemodynamics (heart rate, blood pressure and cardiac contractility) and cardiac pathology^45^. The integrated measurement technologies within this model system (other MEA parameters and flexible cantilevers in combination with a liver module)^6,12^, combined with the ability to collect media samples over time provides the prospect of assessing both changes in cardiac contractility via assessment of force utilizing the flexible cantilevers and pathology via assessment of released soluble cardiac biomarkers, for example, cardiac troponin^46^. Such an approach if validated with a diverse panel of pharmacological agents, and coupled with liver cells of varying metabolic competencies, representing interindividual variability, could enable a holistic assessment of cardiac safety or efficacy in one combined human-based model not only for acute responses, as measured here, but potentially for repeated dose toxicity as cell function can be maintained within these systems for up to 28-days^15^. This would not only allow detection of changes but potentially provide phenotypic fingerprints of individual compounds to be generated.

In conclusion, our findings serve as a guiding principle to future MPS investigations. Whilst these data are robust, we strongly advocate that further compounds, ideally working across different time frames, through other pharmacological mechanisms and representing a broader chemical diversity, should be evaluated to increase the validation of this heart-liver model but also to build mathematical models that enable quantitative translation to animal and human outcome.

## Methods

### Microfluidic housing design and assembly

Microfluidic housings were designed and fabricated as previously described^6,7^ using poly (methyl methacrylate) and PDMS elastomer sheets. The medium recirculates between 2 reservoirs through a microfluidic network formed by the PDMS gaskets, with flow driven by a rocking platform producing a sinusoidal rocking profile with amplitude of ±1° and period of 60 seconds. The medium flows over the cell-containing areas of the liver coverslips and cardiac cantilever and MEA chips within the microfluidic channel. The system contained a total of 2 mL of medium throughout the flow channel and reservoirs. Compound dosing was applied at the reservoir closest to the liver chamber, such that the compound would first pass over and interact with the liver before being recirculated throughout the system.

### Preparation of chips

Cantilever array chips for force measurements were fabricated following protocols previously described^47–52^. Each cantilever chip contained an array of two rows of microscale cantilevers (4 µm thick, 100 µm wide, and 750 µm long), with 16 cantilevers per row. Custom patterned MEA chips, for electrical measurements, were fabricated using standard microfabrication procedures following a previously described protocol^7^. Each MEA chip included 10 platinum recording electrodes separated by 1 mm. The surface of each chip were coated with poly (ethylene glycol) (PEG)-containing silane, and this PEG layer was patterned by selective ablation using a 193 nm ArF excimer laser (Lambda Physik, Santa Clara, CA). The ablated PEG surfaces were coated with human plasma fibronectin (Millipore) diluted in 1X phosphate buffered saline (PBS) (Thermo Scientific) to concentrations of 10 ug/mL for MEAs and 50 ug/mL for cantilevers, then incubated at 37 °C for 30 minutes followed by a PBS rinse. Printed circuit boards and flexible elastomeric connectors (zebra connectors, FUJIPOLY, 1 mm wide × 18.2 mm long × 9 mm tall) were incorporated into each system to create an interface between the MEA and a commercially available 60 electrode amplifier (MEA1060, Multichannel-systems).

### Cell culture

hiPS-CMs (purchased from Cellular Dynamics) were thawed and seeded directly onto the surfaces at 50,000 cells per MEA and 500,000 cells per cantilever^6^. Primary human hepatocytes were purchased from the Cell Resources Core (Massachusetts General Hospital, Boston, MA, USA). Once thawed, 150,000 hepatocytes were cultured in the hepatic chamber which was coated with rat tail collagen type I (60 ug/mL) as previously described^6^. The same batch of primary human hepatocytes and hiPS-CMs were used throughout the course of these experiments to block for any potential batch to batch variation within these cells.

### Drug preparation

Tefenadine (Sigma Aldrich, T9652, St. Louis MO, USA) and the proprietary AstraZeneca compounds AZ12818677 and AZ10565970 were dissolved in DMSO to a concentration 1000x the desired final nominal dosing concentration. Drug was added through the reservoir closest to the liver chamber to allow for first contact of the drug with the liver. 2 μL of the drug stock solution (or vehicle) was added to each system to achieve the desired working concentration in 2 mL of total volume within the platform.

### Cardiac activity as measured by MEA

Custom printed circuit boards were produced to interface the MEAs inside the system with a commercially available 60 electrode amplifier (MEA1060, Multichannel-systems) using a Zebra® elastomeric connector (Fujipoly)^7^. A stimulus generator (STG 1002, Multichannel Systems) was used to stimulate the cells (800 mV rectangular pulse, 0.5 Hz to 3.0 Hz in 0.25 Hz increments). The multichannel systems software suite was used to control both the amplifier and stimulator and was used to record action potentials.

Baseline recordings of spontaneous and stimulated cell activity were taken immediately prior to drug application and at 0.5, 1, 4, 12, 16 and 24 hours after exposure to terfenadine and at 0.5, 1, 2, 4 and 24 hours after exposure to AZ12818677. FPD, spontaneous beat frequency and CV were extracted using Clampfit (Axon Instruments). The FPD was calculated by measuring the time from the initiation of the Q-wave until the apex of t-wave. Spontaneous beat frequency was obtained from spontaneous recordings of cardiac activity 30 seconds in length.

### Immunofluorescence

Hepatocytes and cardiomyocytes were fixed with 4% paraformaldehyde for 15 minutes at room temperature, washed three times with PBS, permeabilized with 0.1% Triton-X for 15 minutes, then blocked with a PBS solution containing 5% donkey serum and 1% BSA for one hour at room temperature. Afterwards, hepatocytes were incubated with sheep anti-human serum albumin primary antibody (Abcam) at a 1:100 dilution overnight at 4 °C, washed 3 times with PBS, then incubated with Alexa Fluor 568 conjugated donkey anti-sheep secondary antibody (Thermo Fisher) at a 1:300 dilution for 2 hours at room temperature. Cardiomyocytes were treated similarly with mouse anti-myosin heavy chain primary antibody (Developmental Studies Hybridoma Bank) at a 1:10 dilution, Alexa Fluor 568 conjugated donkey anti-mouse secondary (Thermo Fisher) at a 1:300 dilution. Both cell types were subsequently stained with DAPI (Thermo Fisher) at a 300 nM concentration for 5 minutes. Cells were imaged on a TCS SP8 confocal microscope (Leica Microsystems) and processed using LAS X software.

### Cell viability

Hepatocyte viability was assessed via a MTT assay. Briefly, cells were incubated in 500 µL of growth medium containing 5 mg/mL MTT powder for 90 minutes at 37 °C, 5% CO_2_. The resultant crystals were dissolved in 100 µL of lysis buffer (10% SDS with 0.5% acetic acid in dimethyl sulfoxide (DMSO)) and was placed into a 96 well plate. Absorbance was read at 570 nm using a BioTek Synergy HT plate reader. Cardiomyocyte viability was assessed using 10% solution (v:v) of alamar blue (Thermofisher) in media at 37 °C, 5% CO_2_ for 4 hours. 100 µL of this solution was then placed into a 96 well plate and read at fluorescence excitation wavelength 570 nm and emission at 590 nm using the BioTeK Synergy HT plate reader.

### Drug determination and quantification with HPLC-MS/MS

Drug concentrations were determined using an LCMS system consisting of an Agilent Technologies (Santa Clara, CA) 1100 HPLC interfaced to an Agilent 6490 Triple Quadrupole Mass Spectrometer with an electrospray inlet. All drugs were separated using gradient elution methodology on a C18 phase (Agilent Zorbax Eclipse Plus, 4.6 mm, 100 mm length, 3 µm particle diameter) with binary mobile phase. Samples (50 µL) were diluted with acetonitrile and centrifuged to precipitate proteins after addition of the internal standard. The MS was set to positive ion mode and multiple reactive monitoring with nitrogen used for collision induced dissociation. The mobile phase used was 20 mM ammonium formate/acetonitrile for terfenadine and fexofenadine separations and 0.1% formic acid/acetonitrile for AZ12818677 and AZ12864610. Terfenadine, fexofenadine, AZ12818677 and AZ12864610 were analyzed with an internal standard, propranolol. For terfenadine, fexofenadine and propranolol quantitation, the MRM (parent/daughter) transitions used were m/z 472 to 436, 502 to 466, and 260 to 116, respectively. The collision energy parameter was set to 24 (terfenadine and fexofenadine) and 16 for propranolol. The MRM (parent/daughter) transitions used for AZ12818677 and AZ12864610 quantitation were m/z 466.5 to 449.0 and 424.4 to 406.3, and the collision energy was set to 20 and 16, respectively. For each of the compounds quantified, the dwell time was set to 50 ms and acceleration voltage to 5 V.

### LC-MS/MS analysis of AZ12818677 and metabolite in cardiomyocyte incubations

hiPS-CMs (iCell™ Cardiomyocytes), cell culture thawing, and maintenance media were purchased from Cellular Dynamics International (cat# CMM-100-120-005) (Madison, WI). hiPS-CMs were cultured on gelatin coated 96 well plates according to the manufacturers protocol http://www.cellulardynamics.com/products/lit/CDI_iCellCMUsersGuide110927.pdf) at a density of 20000 cells/well. Cell culture media was replaced every 48 hours for 10 days. Following 10 days in culture, hiPS-CMs were treated with 0.1% DMSO, AZ12818677 (10, 100 or 200 µM), media and hiPS-CM samples were collated at 0, 2, 6 and 24 hours. Concentration of all analytes in the incubations was determined by LC-MS/MS. Acquity ultra performance liquid chromatography [UPLC] system, (Waters, UK) coupled to a triple-quadrupole mass spectrometer (Xevo TQ-S; Waters, Milford, MA) was used to carry out the sample analysis. The analytes were separated by reverse-phase liquid chromatography using Waters Atlantis® T3, 3 µm, 2.1 × 50 mm column (Waters, UK). Mobile Phases A and B consisted of water (containing 0.1% ammonium formate w/v) and ACN (containing 0.1% FA v/v), respectively. The flow rate was held constant at 0.9 mL/min throughout the gradient run. The initial mobile phase composition of 99% A and 1% B was held for 1 min. Mobile phase B was then increased linearly to 70% until 1.5 min, followed by further increase to 40% B until 3.5 min. At 1.51 min the composition of A and B was made to 1% A and 99% B and was held until 4 minutes. At 4.1 min A and B were reversed to the initial 99% A and 1% B and held for 1 minute. Analytes quantitation was achieved by MS–MS detection in positive electrospray ionization mode. The MS operating conditions were as following: the capillary voltage was 1.14 kV and source offset was 50 V. The desolvation temperature was set to 600 °C. Nitrogen was used as the desolvation gas (800 L/h) and cone gas (150 L/h). Argon was used as the collision gas at a flow rate of 0.15 mL/min. Detection of the ions was performed in the MRM mode using the transitions of m/z 424.21 → 187.98 for AZ12864610 and m/z 455.1984 → 165.2068 for verapamil, the internal standard, with a Dwell time of 0.03, 0.05 and 0.02 s, respectively. Peak integration and calibrations were performed using TargetLynx software (Version 4.1, Waters, Milford, MA).

### hERG inhibition electrophysiology

A functional electrophysiology assay in CHO cells stably transfected with Kv11.1 (hERG) was conducted on the IonWorks™ platform as described previously^53^. AZ12818677 and AZ12864610 were tested at 0.01, 0.03, 0.1, 0.33, 1, 3, 10, 33 and 100 µM in 0.33% DMSO. Concentration-effect curves were plotted for each compound and expressed relative to vehicle (0.33% DMSO) ± SD. The molar concentration of test compound producing 50% inhibition (IC_50_) were calculated using Graphpad Prism™ (La Jolla, CA) to fit data to a 4-parameter non-linear regression curve. The maximum (DMSO control) values were fixed. IC_50s_/EC_50s_ were generated in quadruplet for each compound and geometric means and 95% CI were subsequently calculated.

### Anesthetized Guinea pig

This study was ethically reviewed by the establishments’ Animal Welfare and Ethical Review Body (AWERB), following the principles of the 3Rs. All work was then conducted under an approved project license, governed by the UK home office under The Animals (Scientific Procedures) Act 1986. All animal identification, conditions of housing, acclimatization, environment, diet and water were in accordance with the standard AstraZeneca operating procedures. The effects on cardiac electrophysiology and bioanalysis were assessed in the anaesthetized Guinea pig (Dunkin Hartley) via recording of the ventricular monophasic action potential at 90% repolarization (MAPD90) as described previously^54^. AZ12818677 or vehicle was tested (I.V.) at 0.744 and 2.015 mg/kg (4 animals per group), formulated as a solution in 30% v/v dimethylacetamide in water (vehicle). Recordings were obtained for 20 minutes following the start of each dose level followed by a 30-minute washout.

### Heart only and heart-liver chip PKPD modelling and *in vivo* simulation

Time course terfenadine and fexofenadine concentration profiles were modelled using a 4-compartment model in Phoenix NLME Software version 7.0. Heart, liver, media, and PDMS compartments and their corresponding parameters can be seen outlined in Fig. 4a. Flow of terfenadine for medium-PDMS and medium-heart pathways was modelled using a first order process as defined by parameters KP1/KP2 and KL1/KL2, respectively. Flow of terfenadine from medium and heart compartments was assumed to be saturable as defined by Eqs 1 and 2. Metabolism of terfenadine to fexofenadine within the heart and liver compartments was modelled using a first order process as defined by parameters Kfc and Kfl respectively. It was assumed that there was a delay of fexofenadine release into the system as defined by parameter Kint.

A fifth compartment, FPD, was added to the Phoenix model to represent a PD profile linked to the defined PK model. This PD profile was modelled as a first order process in which the change in effect on FPD was a function of terfenadine heart bioaccumulation as defined by parameters K_FPDin_ and K_FPDout_. An indirect modelling approach was used for FPD because a hysteresis relationship was found between cardiac terfenadine concentration and FPD values (Supplementary Fig. 1). This approach was further confirmed when %CV values increased when modelled directly.

Using the experimental data collected (Fig. 3), parameters (except for Kint) were simultaneously fit across heart only and heart-liver systems. Parameters were then fixed and the fexofenadine parameter, Kint, was fit using the experimental fexofenadine concentration data collected (Fig. 3). In the heart only case, the liver compartment was eliminated resulting in relevant parameters being equal to 0. Volumes of each compartment was defined by system parameters described above. The liver volume was initialized based on the system estimates but allowed to vary to account for any differences between assumed and actual cell numbers or sizes. Once parameters were estimated, the model was used to simulate FPD profiles given published *in vivo* terfenadine PK profiles. Published Guinea pig^23^, dog^24^ and human^25^, PK profiles were used as inputs into the established model in order to predict cardiac bioaccumulation over time, and therefore predict change in predicted FPD. While Guinea pig and dog temporal PKPD data is available in the literature, matching human data is not. Instead, the model made use of a hypothesized dosing schedule that would simulate a PK profile matching published Cmax and AUC data in humans orally dosed with terfenadine, as this information is available in the literature^25^. The reported standard deviation values in PK profile were used to capture the associated error in predicted FPD values. Percent free protein in plasma was assumed to be 2% for dog and 3% for Guinea pig and human for the FPD simulation^23,24^.

1 $${{\boldsymbol{K}}}_{{\boldsymbol{medium}}{\boldsymbol{to}}{\boldsymbol{heart}}}={\boldsymbol{KC}}{\bf{1}}$$

2 $${{\boldsymbol{K}}}_{{\boldsymbol{heart}}{\boldsymbol{to}}{\boldsymbol{medium}}}={\boldsymbol{KC}}{\bf{2}}\ast (\frac{{\bf{1}}}{{\bf{1}}+\frac{{\boldsymbol{Cardia}}{{\boldsymbol{c}}}_{{\boldsymbol{Terf}}}}{{\boldsymbol{I}}{{\boldsymbol{C}}}_{{\bf{50}}}}})$$

## Supplementary information

Supplementary Dataset 1

## Data Availability

All data supporting the findings of this study are available within the article and its Supplementary Information Files.
