# Supplementary material for: On the potential of in vitro organ-chip models to define temporal pharmacokinetic-pharmacodynamic relationships
Source: Sci Rep. 2019 Jul 3;9:9619. doi: 10.1038/s41598-019-45656-4 (PMC6610665; doi:10.1038/s41598-019-45656-4)
Supplement: Supplementary file 1 — Supplementary Dataset 1 [file 41598_2019_45656_MOESM1_ESM.docx]

**On the potential of *in vitro* organ-chip models to define temporal pharmacokinetic-pharmacodynamic relationships**

Christopher W. McAleer^1^†, Amy Pointon^2^†, Christopher J. Long^1^, Rocky L. Brighton^1^, Benjamin D. Wilkin^1^, L. Richard Bridges^1^, Narasimhan Narasimhan Sriram^1^, Kristin Fabre^3^, Robin McDougall^3^, Victorine P. Muse^3^, Jerome T. Mettetal^3^, Abhishek Srivastava^2^, Dominic Williams^2^, Mark T. Schnepper^4^, Jeff L. Roles^1^, Michael L. Shuler^1^, James J. Hickman^1,4^, Lorna Ewart^2^*

Affiliations

^1^ Hesperos, Inc., 3259 Progress Dr., Room 158, Orlando, FL 32826-3230, US

^2^ Drug Safety and Metabolism, IMED Biotech Unit, AstraZeneca, Cambridge, UK

^3^ Drug Safety and Metabolism, IMED Biotech Unit, AstraZeneca, Waltham, US

^4^ NanoScience Technology Center, 12424 Research Parkway, Suite 400, Orlando, FL 32826, US

* Corresponding author (lorna.ewart@astrazeneca.com)

† These authors contributed equally to this work

# Enquires relating to Hesperos should be sent to jhickman@hesperosinc.com


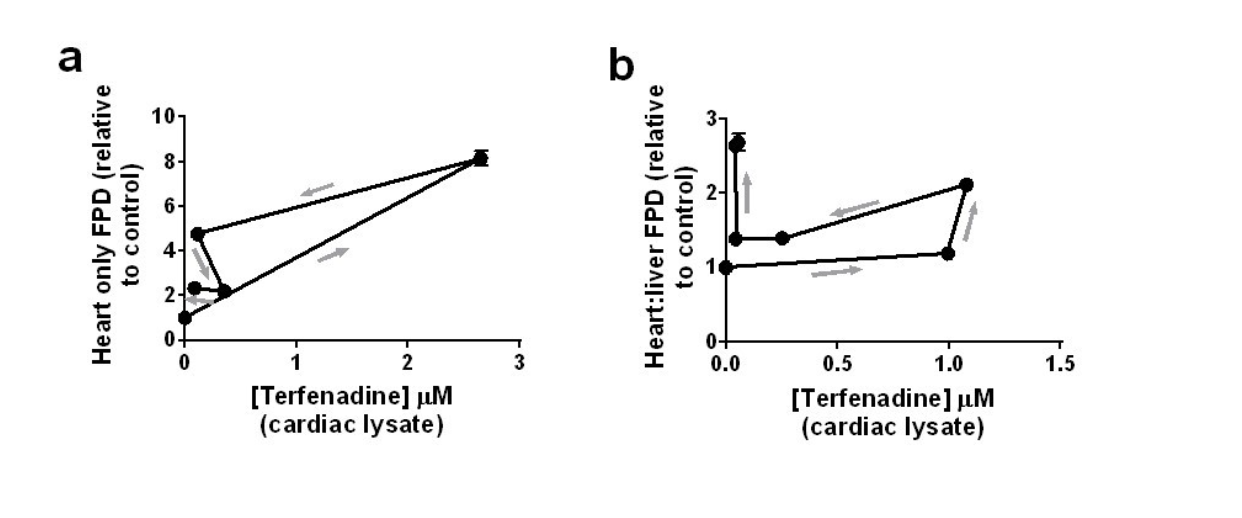


**
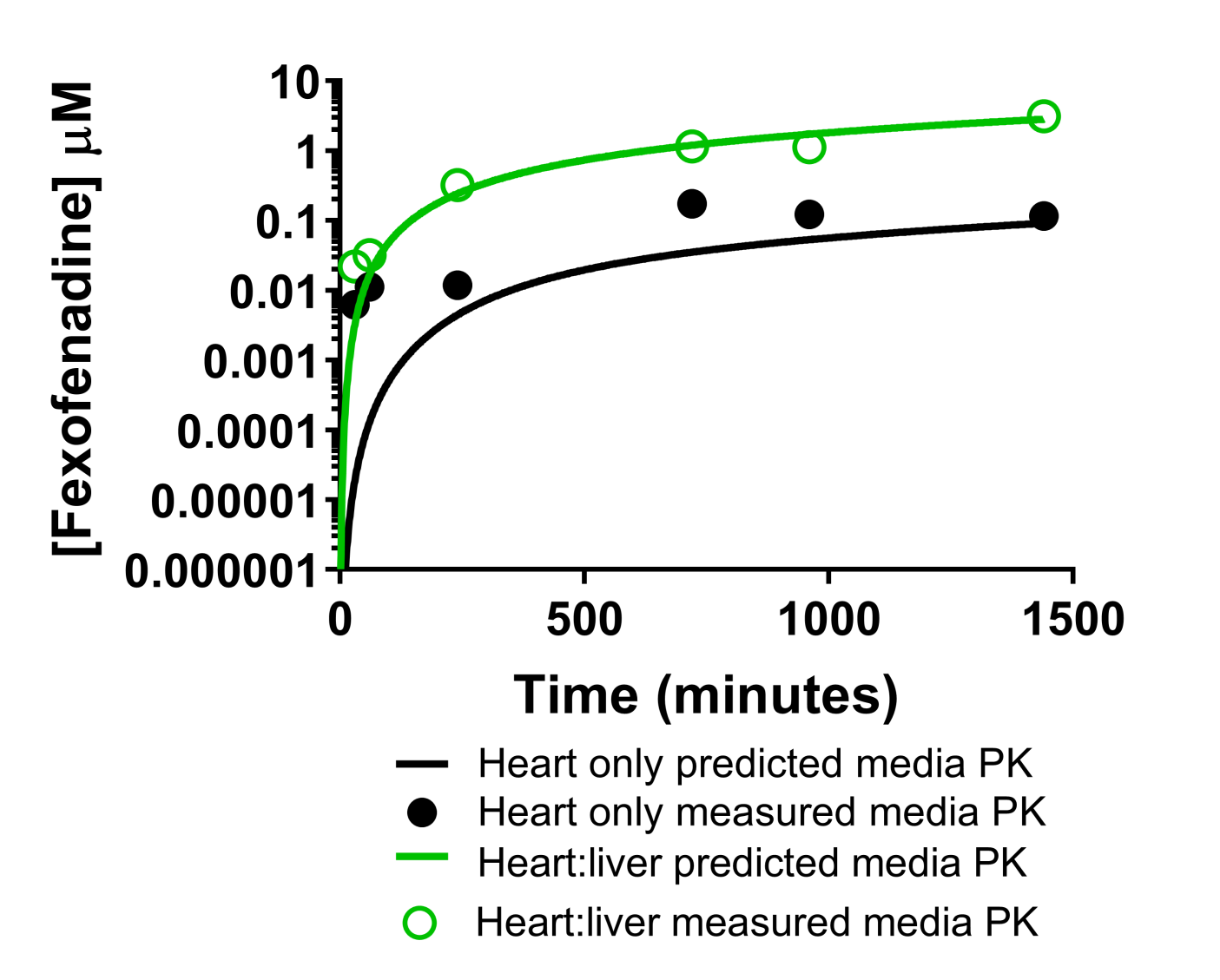
**

**Supplementary Information**

**Supplementary Figure legends**

**Supplementary Figure 1:** Hysteresis loops of observed cardiac lysate concentration (n=2) and corresponding observed FPD data (n≥3) for heart only (a) and heart: liver (b) systems (Mean ± SD). The grey arrows represent the progression of time.

**Supplementary Figure 2:** PK profiles (solid line) and observed data (closed circle) for total fexofenadine over 24 hours are modelled in both heart only (black) and heart:liver (green) models.

**Supplementary Table 1:** parameter estimates, and associated error values generated by Phoenix NLME 7.0 software.

**Supplementary information: Phoenix code**

test(){

**### Heart:Liver Chip PK model**

deriv(C_pdms = KP1*C_media - KP2*C_pdms)

deriv(C_cardiac = KC1*C_media - KC2*(1/(1+(C_cardiac/IC50)))*C_cardiac - Kfc*C_cardiac)

deriv(C_liver = KL1*C_media - KL2*C_liver - Kfl*C_liver)

deriv(C_media = -1*(KP1 + KC1 + KL1)*C_media + KP2*C_pdms + KC2*(1/(1+(C_cardiac/IC50)))*C_cardiac + KL2*C_liver)

deriv(C_fexo = Kfl*C_liver + Kfc*C_cardiac )

dosepoint(C_media)

**### Heart:Liver chip PD model**

deriv(AE_CL = kon*C_cardiac - koff*AE_CL)

FPD_CL = 1 +AE_CL

**### Heart:Liver error statements**

error(CEps_cardiac = 0.00642974)

observe(CObs_cardiac = C_cardiac + CEps_cardiac)

error(CEps_liver = 0.0101382)

observe(CObs_liver = C_liver + CEps_liver)

error(CEps_media = 1.17403)

observe(CObs_media = C_media + CEps_media)

error(CEps_fexo = 1.84938)

observe(CObs_fexo = C_fexo + CEps_fexo)

error(FPDEps_CL = 0.995963)

observe(FPDObs_CL = FPD_CL + FPDEps_CL)

**### Heart only chip PK model**

deriv(C_pdmsC = KP1*C_mediaC - KP2*C_pdmsC)

deriv(C_cardiacC = KC1*C_mediaC - KC2*(1/(1+(C_cardiacC/IC50)))*C_cardiacC - Kfc*C_cardiacC)

deriv(C_mediaC = -1*(KP1 + KC1)*C_mediaC + KP2*C_pdmsC + KC2*(1/(1+(C_cardiacC/IC50)))*C_cardiacC)

deriv(C_fexoC = Kfc*C_cardiacC)

dosepoint(C_mediaC)

**### Heart only chip PD model**

deriv(AE_C = kon*C_cardiacC - koff*AE_C)

FPD_C = 1 +AE_C

**### Heart only error statements**

error(CEps_cardiacC = 0.01338)

observe(CObs_cardiacC = C_cardiacC + CEps_cardiacC)

error(CEps_mediaC = 1.07091)

observe(CObs_mediaC = C_mediaC + CEps_mediaC)

error(CEps_fexoC = 0.0323472)

observe(CObs_fexoC = C_fexoC + CEps_fexoC)

error(FPDEps_C = 0.38903)

observe(FPDObs_C = FPD_C + FPDEps_C)

**### Parameter values**

KP1 = 0.0035097

KP2 = 0.000526081

KC1 = 0.00185601

KC2 = 0.0780807

KL1 = 0.0256725

KL2 = 0.0429636

Kfl = 0.0167355

Kfc = 0.000912019

IC50 = 0.0699277

kon = 0.147464

koff = 0.00413283

}

**Supplementary Table 1**

| **Parameter** | **Estimate** | **Units** | **Stderr** | **CV%** | **2.5% CI** | **97.5% CI** | **Var. Inf. factor** |
| --- | --- | --- | --- | --- | --- | --- | --- |
| KP1 | 0.005 | 1/min | 0.00045 | 12.9307 | 0.00259 | 0.00443 | 1.36E-06 |
| KP2 | 0.00052 | 1/min | 0.00013 | 25.4337 | 0.00025 | 0.0008 | 1.18E-07 |
| KC1 | 0.0019 | 1/min | 0.00012 | 6.37414 | 0.00162 | 0.0021 | 9.25E-08 |
| KC2 | 0.04698 | 1/min | 0.01475 | 18.8854 | 0.04811 | 0.10805 | 0.00144 |
| KL1 | 0.03749 | 1/min | 0.00142 | 5.526 | 0.02279 | 0.02856 | 1.33E-05 |
| KL2 | 0.04988 | 1/min | 0.00405 | 9.41983 | 0.03474 | 0.05119 | 0.00011 |
| Kfl | 0.0162 | 1/min | 0.00086 | 5.1621 | 0.01498 | 0.01849 | 4.93E-06 |
| Kfc | 0.0009 | 1/min | 0.00014 | 15.0636 | 0.00063 | 0.00119 | 1.25E-07 |
| IC50 | 0.10493 | µmol | 0.01571 | 22.4722 | 0.03799 | 0.10186 | 0.00163 |
| Kon | 0.13751 | 1/min | 0.01329 | 9.00971 | 0.12046 | 0.17446 | 0.00117 |
| Koff | 0.00388 | 1/min | 0.00057 | 13.678 | 0.00298 | 0.00528 | 2.11E-06 |
| tvKint | 0.00062 | n/a | 4.9E-05 | 7.96347 | 0.00051 | 0.00073 | 5.7E-07 |
| tvV_Liver | 1.49995 | n/a | 0.11963 | 7.97551 | 1.25628 | 1.74362 | 9.39E-02 |

**Supplementary Text 1:** Transcript of code used in Phoenix NLME to define PKPD mathematical model for the MPS chip. The code outlined the heart only and heart:liver chip separately to allow for parameters to be fit simultaneously.
